# Synthesis of amorpha-4,11-diene from dihydroartemisinic acid

**DOI:** 10.1016/j.tet.2018.12.050

**Published:** 2019-02-08

**Authors:** Geoffrey Schwertz, Andrea Zanetti, Marllon Nascimento de Oliveira, Mario Andrès Gomez Fernandez, Fabienne Dioury, Janine Cossy, Zacharias Amara

**Affiliations:** a Laboratoire de Chimie Organique, Institute of Chemistry, Biology and Innovation (CBI), UMR 8231, ESPCI ParisTech/CNRS/PSL Research University, Paris Cedex 05, France; b Equipe de Chimie Moléculaire, Laboratoire GBCM, EA7528, Conservatoire National des Arts et Métiers, HESAM Université, 2 rue Conté, 75003, Paris, France

**Keywords:** Amorphadiene, Artemisinin, Malaria, Elimination, Microwaves

## Abstract

Amorphadiene is a natural product involved in the biosynthesis of the antimalarial drug artemisinin. A convenient four-step synthesis of amorphadiene, starting from commercially available dihydroartemisinic acid, is reported. The targeted molecule is isolated with an overall yield of 85% on a multi-gram scale in four steps with only one chromatography.

## Highlights

• A direct method for the production of amorpha-4,11-diene.
• The multi-step sequence is high yielding, scalable and reproducible.
• The multi-step sequence involves only one chromatography.
• The last step is accelerated using microwave irradiation.

## Graphical abstract

**Figure:**
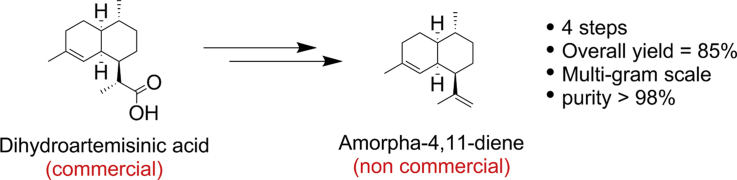

## 1 Introduction

**Scheme 1:**
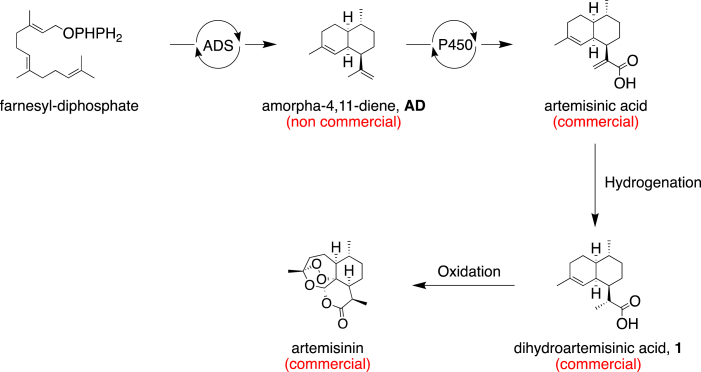
Semi-synthetic approach for the production of artemisinin (Amyris/Sanofi processes).

Amorphadiene (**AD**) is produced in plants by cyclization of farnesyl-pyrophosphate by the enzyme amorphadiene synthase (ADS) (Scheme 1) [1]. **AD** is a key intermediate in the biosynthesis of the antimalarial drug artemisinin [1,2]. In this context, the synthesis of **AD** has been described using a fermentation route (Amyris process) [[2], [3], [4]]. However, **AD** is not yet commercially available. Herein, we report a short and high-yielding gram-scale synthesis of **AD** starting from the commercially available dihydroartemisinic acid, **1**, which is an intermediate in the Sanofi process to prepare artemisinin [5].

**Scheme 2:**
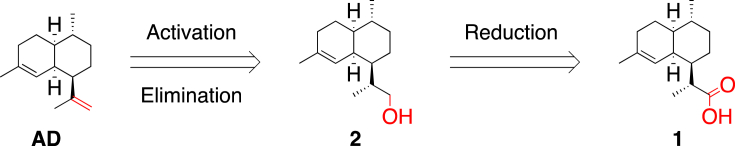
Formation of amorphadiene, **AD**, from dihydroartemisinic acid, **1**.

With the goal of providing a direct and scalable access to **AD** from the commercially available natural product dihydroartemisinic acid, **1**, a three-step synthetic procedure, relying on the carboxylic acid reduction to the corresponding alcohol **2**, followed by an activation/elimination sequence to produce **AD**, was envisioned (Scheme 2). Bouwmeester et al. described a similar synthetic approach as ours, starting from artemisinic acid, affording **AD** with an overall yield of 25% [6]. However, only a generic route was reported without detailed procedures, scale, and yield for each individual step.

## 2 Results and discussion

Our attempts for the direct reduction of **1** to **2** focused on the use of lithium aluminium hydride (LiAlH_4_) as a reducing agent. The reaction was first tested using 2.0 equivalents of LiAlH_4_ in anhydrous THF at 0 °C (Table 1, entry 1) [7]. However, only moderate conversion of **1** was obtained (60%) and alcohol **2** was isolated in only 25% yield after purification. By increasing the amount of LiAlH_4_ in freshly distilled Et_2_O and, after stirring at 23 °C for 2 h, alcohol **2** was obtained with a good yield of 85% without purification (Table 1, entry 2). This yield was further improved to 95% by stirring the reaction mixture for 24 h (Table 1, entry 3). Ultimately, we were able to optimize these conditions using 3.0 equivalents of LiAlH_4_ in non-distilled Et_2_O at a concentration of 0.29 M (Table 1, entry 4).

**Table 1:**
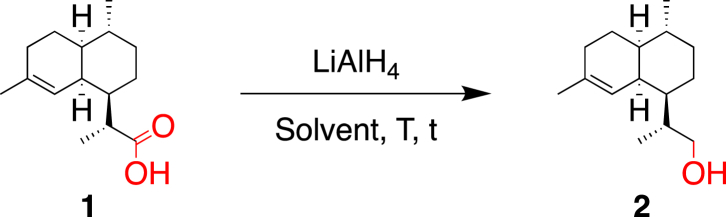
Reduction of **1** to alcohol **2**.

Entry	LiAlH_4_ (equiv)	Solvent	T	t [h]	Conversion	Yield^a^
1	2.0	THF (0.18 M)	0 °C	1	ca. 60%	25%
2	5.0	Et_2_O (0.07 M)	0 °C → 23 °C	2	100%	85%
3	5.0	Et_2_O (0.07 M)^b^	0 °C → 23 °C	24	100%	95%
4	3.0	Et_2_O (0.29 M)^b^	0 °C → 23 °C	16	100%	95%

a Isolated yields.

b The reaction was conducted with non-distilled solvent.

**Scheme 3:**
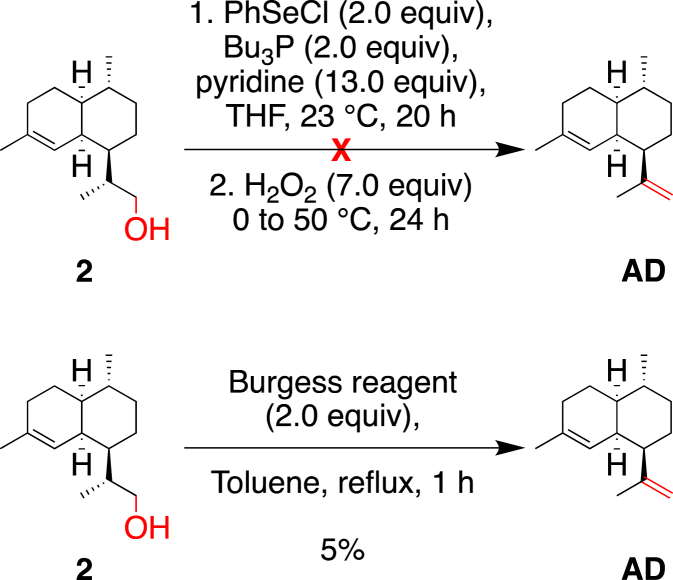
Attempts for the direct synthesis of **AD** from alcohol **2**.

Different conditions were then evaluated to attempt direct conversion of **2** into **AD**. Unfortunately, the direct elimination of the hydroxyl group, using either the Burgess reagent [8] or a one-pot selenide strategy, inspired in the Grieco method [9], failed to give satisfying results even on purified **2** (Scheme 3) [10].

**Scheme 4:**
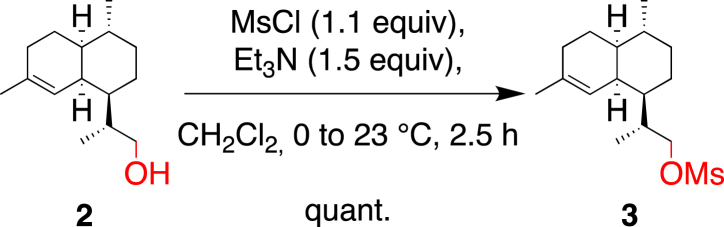
Transformation of alcohol **2** to the corresponding mesylate **3**.

Alternatively, we considered the activation of the alcohol followed by an elimination. Alcohol **2** was quantitatively converted to its corresponding mesylate **3** (MsCl, 1.1 equiv; Et_3_N, 1.5 equiv; in anhydrous CH_2_Cl_2_, 1 M) (Scheme 4) [11]. Bouwmeester et al. reported the same transformation of **2** to **3** using pyridine as solvent and base, which however, required purification by column chromatography to isolate pure mesylate **3** [6]. We replaced pyridine with dichloromethane as solvent and used only 1.5 equivalents of base, which afforded pure **3** without further purification.

The elimination of the leaving group and formation of the C=C double bond turned out to be more challenging than anticipated. Several conditions were attempted based on related literature procedures (Table 2) [[12], [13], [14]]. As the direct elimination of the mesylate group failed to give satisfying results (Table 2, entry 1), **3** was treated with sodium iodide (NaI, 5.0 equiv) and 1,8-diazabicyclo[5.4.0]undec-7-ene (DBU, 3.0 equiv) in a one-pot reaction, upon which **AD** was isolated, after column chromatography, in 35% yield (Table 2, entry 2). Alternatively, DBU was added only after completion of the Finkelstein reaction using NaI (2.0–10.0 equiv) but yields in **AD** remained moderate (30–40%; Table 2, entries 3–5).

**Table 2:**
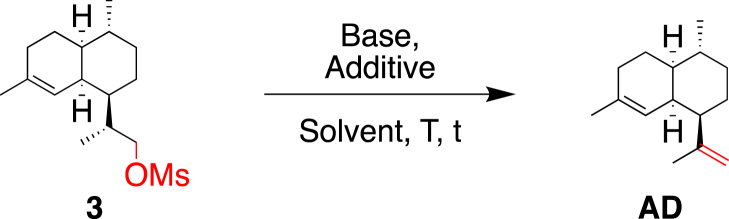
Primary screening experiments for the elimination step.

Entry	Base (equiv)	Additive (equiv)	“steps”	Solvent	T	t [h]	Conversion	Yield
1	DBU (3.0)	–	1	THF	reflux	20	0%	–
2	DBU (3.0)	NaI (5.0)	1	DMF	80 °C → 23 °C	48	100%	35%
3	DBU (3.0)	NaI (2.0)	2	DME	60 °C → reflux	5 then 3	100%	30%
4	DBU (5.0)	NaI (5.0)	2	Acetone	Reflux	2.5 then 13	100%	40%
5	DBU (5.0)	NaI (10.0)	2	Acetone	Reflux	13	100%	30%
				THF	65 °C	3.5		

**Scheme 5:**
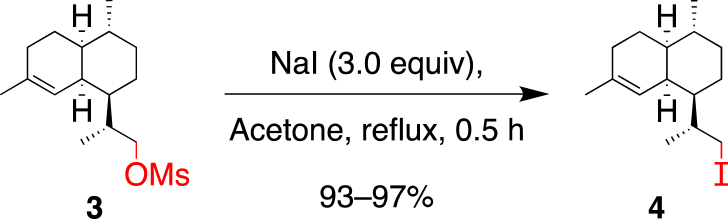
Conversion of mesylate **3** into the iodinated intermediate **4**.

As the one-pot procedure was not high yielding, another method used by Baran et al. on a similar scaffold as **3**, [15] consisting of isolating the iodo intermediate **4**, after reacting **3** with NaI prior to the addition of base was realized (Scheme 5). The substitution of the mesylate moiety by an iodine proceeded well, affording **4** in yields to 97%.

After isolation, the iodo-compound **4** was reacted with DBU (5.0 equiv) in boiling acetone and **AD** was isolated in 45% yield (Table 3, entry 1). It is worth noting that 4-hydroxy-4-methylpentan-2-one was also isolated from this reaction, which presumably indicates that a self-condensation of acetone occurred and thus, acetone was not considered as a relevant solvent to carry out this transformation. In THF, a longer reaction time (24 h instead of 5 h) was required to achieve full conversion of **4** (Table 3, entry 2) but the yield in **AD** remained modest (45%). With these results in hand, we postulated that DBU could promote undesirable side reactions and we decided to use alternative bases. No conversion was observed with Et_3_N (5.0 equiv) (Table 3, entry 3), whereas a moderate conversion was recorded with 5.0 equivalents of *t*-BuOK after 40 h at 65 °C (55%) (Table 3, entry 4). This result was improved by using microwave irradiation and gratifyingly, a complete conversion of **4** was obtained after 1 h producing **AD** in 89% yield (Table 1, entry 5). Reducing the amount of *t*-BuOK to 3.0 equiv did not give full conversion of **4** after 1 h in THF (Table 3, entry 6). By using a freshly prepared solution of *t*-BuOK in THF (1 M), the excess of base was reduced to 1.3 equivalents and the reaction time to 0.5 h, affording **1** in an excellent yield of 96% (Table 3, entry 8). More importantly, it allowed us to increase the concentration in **4** to 0.75 M, which also enabled to scale-up the reaction to 4 g of **4** per batch using a standard 20 mL sealed tube.

**Table 3:**
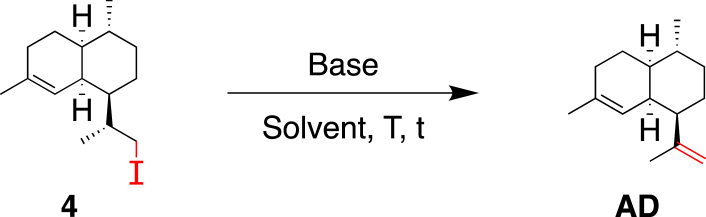
Optimization of the conditions for the elimination step.

Entry	Base (equiv)	Solvent	[C]	T [°C]	Time [h]	Conversion	Yield
1	DBU (5.0)	Acetone	0.15 M	Reflux	5	100%	45%^a^
2	DBU (5.0)	THF	0.15 M	65	24	100%	45%^a^
3	Et_3_N (5.0)	THF	0.15 M	65	40	0%	–
4	*t*-BuOK (5.0)	THF	0.15 M	65	40	55%	47%^b^
5	*t*-BuOK (5.0)	THF	0.15 M	65 (*μ*W)^c^	1	100%	89%^a^
6	*t*-BuOK (3.0)	THF	0.15 M	65 (*μ*W)	1	55%	47%^b^
7	*t*-BuOK (6.7) (1 M in THF)		0.15 M	65 (*μ*W)	0.5	100%	90%^b^
8	*t*-BuOK (1.3) (1 M in THF)		0.75 M	65 (*μ*W)	0.5	100%	96%^a^
9	*t*-BuOK (5.0)	*t*-BuOH	0.15 M	65	5	50%	48%^b^
10	*t*-BuOK (5.0)	*t*-BuOH	0.15 M	90	5	100%	86%^b^
11	*t*-BuOK (2.0)	*t*-BuOH	1.50 M	90	3.5	100%	63%^a^

a Isolated yield.

b Estimated yield based on the GC/MS chromatogram of crude product.

c *μ*W = microwave irradiation.

Because thermal heating is usually preferred over microwave-assisted reaction for large scale synthesis, we also investigated the preparation of **AD** using conventional heating. A moderate conversion (50%) of **4** was achieved when the reaction was performed in *tert*-butanol (*t*-BuOH) at 65 °C (Table 3, entry 9), whereas the iodo intermediate **4** was fully converted to **AD** when the reaction was carried out in boiling *t*-BuOH (Table 3, entry 10). Ultimately, we were able to perform the elimination with only 2.0 equivalents of *t*-BuOK (on a 6.5 g scale) and to isolate **AD** in 63% yield (Table 3, entry 11). We found that, in this case, 10% of the alcohol **2** was formed along with other unidentified side products (estimated yield based on ^1^H NMR).

## 3 Conclusion

**Scheme 6:**
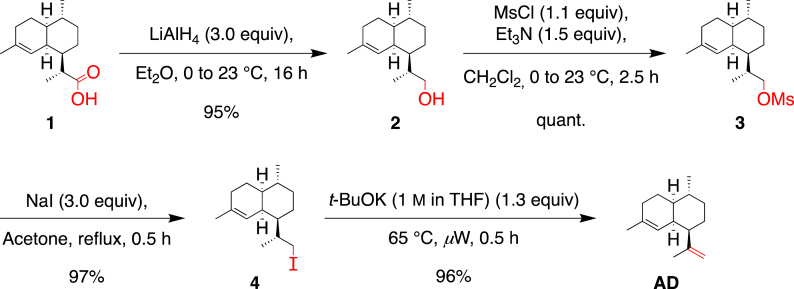
Synthesis of amorphadiene, **AD**, from dihydroartemisinic acid, **1**.

In summary, a screening of conditions allowed us to secure a straightforward access to the important terpene, amorphadiene (**AD**), from commercially available dihydroartemisinic acid **1** (Scheme 6). The most challenging step was the elimination step, which required considerable optimization. Eventually, 1.3 equiv of *t*-BuOK (1 M in THF) were sufficient to achieve full conversion of **4** in 0.5 h and to produce **AD** in 96% yield. Alternatively, we also developed a thermal approach in boiling *t*-BuOH affording **AD** in 63% yield. It is noteworthy to mention that alcohol **2** could be transformed in one step to its corresponding bromo derivative **5** by employing an Appel bromination (see Experimental Section) [16], however, a lower yield (35%) was obtained for the elimination step.

A reliable and straightforward multi-gram scale synthesis of amorphadiene from dihydroartemisinic acid has been established with an overall yield of 85%. This will enable future scale-up and access to this key molecule for studying its transformation into artemisinic acid and artemisinin derivatives.

## 4 Experimental section

### 4.1 General information

Reagents (Aldrich) were purchased as reagent grade and used without further purification. Reactions in the absence of air and moisture were performed in oven-dried glassware under Ar atmosphere. Flash column chromatography was performed using SiO_2_ (60 Å, 230–400 mesh, particle size 0.040–0.063 mm, Merck) at 23 °C with a head pressure of 0.0–0.5 bar. The solvent compositions are reported individually in parentheses. Analytical thin layer chromatography (TLC) was performed on aluminium sheets coated with silica gel 60 F254 (Merck, Macherey-Nagel) or with silica gel 60 RP-18 F_254s_ (Merck, Macherey-Nagel). Visualization was achieved using an alkaline aqueous solution of potassium permanganate (KMnO_4_). Evaporation *in vacuo* was performed at 25–35 °C and 900–10 mbar. Reported yields refer to spectroscopically and chromatographically pure compounds that were dried under high vacuum (0.1–0.05 mbar) before analytical characterization. ^1^H and ^13^C nuclear magnetic resonance (NMR) spectra were recorded on a Bruker AV 400 spectrometer at 400 MHz (^1^H) and 101 MHz (^13^C). Chemical shifts *δ* are reported in ppm upfield using the residual deuterated solvent signals as an internal reference (CDCl_3_: *δ*_H_ = 7.26 ppm, *δ*_C_ = 77.16 ppm). For ^1^H NMR, coupling constants *J* are given in Hz and the resonance multiplicity is described as s (singlet), d (doublet), t (triplet), q (quartet), m (multiplet), and br (broad). All spectra were recorded at 298 K. Infrared (IR) spectra were recorded on a Bruker Tensor 27 FT-IR spectrometer and are reported as wavenumbers $\tilde{\nu }$(cm^−1^). High-resolution mass spectrometry (HRMS) was performed by the Laboratoire de Spectrométrie de Masse from Sorbonne Université, Paris. Gas Chromatography coupled to Mass Spectrometry (GC/MS) analysis was performed on a Shimadzu GCMS-QP2010S using an electronic impact (EI) spectrometer. Low-resolution mass spectra (LRMS) result from ionization by electronic impact (EI-LRMS). The abundance indicated for each mass number (*m/z* values) is given in percentage relative to the strongest peak of 100% abundance (base peak). Melting points were determined using a Büchi melting point apparatus in open capillaries. Nomenclature follows the suggestions proposed by the software ChemDraw Professional 2016.

### 4.2 Synthesis of amorpha-4,11-diene (**AD**)

#### 4.2.1 (R)-2-((1*R*,4*R*,4a*S*,8a*S*)-4,7-Dimethyl-1,2,3,4,4a,5,6,8a-octahydronaphthalen-1-yl)propan-1-ol (**2**)

A suspension of LiAlH_4_ (12.2 g, 321.5 mmol) in Et_2_O (120 mL) was cooled below 0 °C with an ice/sodium chloride/water bath and treated over 1.5 h with a solution of dihydroartemisinic acid (**1**) (25.0 g, 105.78 mmol) in Et_2_O (250 mL) while maintaining the internal temperature below 7 °C. The mixture was maintained in the cold bath, allowed to warm up to 23 °C, stirred for 16 h, cooled below 0 °C, and treated dropwise with H_2_O (12.5 mL; 1.0 mL/g of LiAlH_4_) while maintaining the internal temperature below 5 °C. The mixture was stirred at 5 °C for 20 min, treated carefully with a 15% NaOH solution in H_2_O (12.5 mL; 1.0 mL/g of LiAlH_4_), stirred for 10 min at 5 °C, and treated with H_2_O (37.0 mL; 3.0 mL/g of LiAlH_4_) while maintaining the internal temperature below 5 °C. Anhydrous MgSO_4_ was added to absorb the remaining water and the mixture was filtered. The filtrate was evaporated to afford **2** (22.40 g, 95%) as a white solid.

m.p. 83.9–84.3 °C; *R*_f_ = 0.22 (SiO_2_; petroleum ether/EtOAc 95:05; KMnO_4_); [α]_D_^20^ = −8.8° (*c* 1.04, CHCl_3_); ^1^H NMR (400 MHz, CDCl_3_): *δ* = 5.21 (br s, 1H), 3.75 (m, 1H), 3.52 (m, 1H), 2.47 (br s, 1H), 2.08–1.72 (m, 3H), 1.69–1.57 (m, 3H), 1.61 (s, 3H), 1.53 (m, 1H), 1.41 (m, 1H), 1.30–1.14 (m, 2H), 1.00 (d, *J* = 6.8 Hz, 3H), 1.08–0.88 (m, 2H), 0.86 ppm (d, *J* = 6.4 Hz, 3H), OH signal too weak to be observed; ^13^C NMR (101 MHz, CDCl_3_): *δ* = 135.3, 120.8, 67.0, 42.8, 42.2, 37.7, 36.8, 35.8, 27.8, 26.8, 26.5, 26.0, 24.0, 20.0, 15.1 ppm; IR (ATR): $\tilde{\nu }$ = 3335, 3328, 2920, 2865, 1467, 1451, 1432, 1370, 1025, 991, 955 cm^−1^; HR-ESI-MS: *m/z*: 245.1875 ([*M* + Na]^+^, calcd for C_15_H_26_ONa^+^: 245.1876); GC/MS: *m/z* (%): 222 (11, [*M*]^+^), 191 (14, [*M* – CH_3_O]), 163 (100, [*M* – C_3_H_7_O]^+^), 149 (10), 135 (15), 121 (25), 107 (36), 105 (14). Analytical data correspond to the literature [17].

#### 4.2.2 (R)-2-((1*R*,4*R*,4a*S*,8a*S*)-4,7-Dimethyl-1,2,3,4,4a,5,6,8a-octahydronaphthalen-1-yl)propyl methanesulfonate (**3**)

A solution of **2** (18.89 g, 85.0 mmol) in anhydrous CH_2_Cl_2_ (85 mL) was cooled to 0 °C, treated with Et_3_N (18.4 mL, 127.5 mmol), stirred for 5 min at 0 °C, and treated dropwise with MsCl (7.6 mL, 93.5 mmol) leading to a white precipitate. The mixture was stirred at 23 °C for 2.5 h and diluted with CH_2_Cl_2_ (40 mL). The organic layer was washed with 1 N HCl (2 × 20 mL), dried over MgSO_4_, filtered, and evaporated to afford mesylate **3** (25.64 g, quant.) as a white crystalline solid.

m.p. 71.7–73.5 °C; *R*_f_ = 0.24 (SiO_2_; petroleum ether/EtOAc 95:05; KMnO_4_); [α]_D_^20^ = −9.8° (*c* 1.02, CHCl_3_); ^1^H NMR (400 MHz, CDCl_3_): *δ* = 5.14 (br s, 1H), 4.29 (dd, *J* = 9.5, 3.2 Hz, 1H), 4.12 (dd, *J* = 9.5, 6.2 Hz, 1H), 3.00 (s, 3H), 2.47 (br s, 1H), 2.00–1.72 (m, 4H), 1.69–1.48 (m, 3H), 1.63 (s, 3H), 1.40 (m, 1H), 1.31–1.16 (m, 2H), 1.04 (d, *J* = 6.8 Hz, 3H), 1.02–0.87 (m, 2H), 0.86 ppm (d, *J* = 6.5 Hz, 3H); ^13^C NMR (101 MHz, CDCl_3_): *δ* = 135.9, 120.0, 74.1, 42.5, 42.0, 37.4, 37.3, 35.5, 34.6, 27.7, 26.8, 26.3, 25.9, 24.0, 19.8, 15.2 ppm; IR (ATR): $\tilde{\nu }$ = 2911, 2868, 1449, 1353, 1175, 1110, 953 cm^−1^; HR-ESI-MS: *m/z*: 323.1650 ([*M* + Na]^+^, calcd for C_16_H_28_O_3_SNa^+^: 323.1651); GC/MS: *m/z* (%): 300 (11, [*M*]^+^), 163 (100, [*M* – C_4_H_9_O_3_S]^+^), 147 (17), 133 (10), 121 (29), 119 (19), 107 (31), 105 (20).

#### 4.2.3 (1*R*,4*R*,4a*S*,8a*S*)-1-((R)-1-Iodopropan-2-yl)-4,7-dimethyl-1,2,3,4,4a,5,6,8a-octahydronaphthalene (**4**)

A solution of mesylate **3** (12.03 g, 40.0 mmol) in acetone (80 mL) was treated with NaI (18.0 g, 120.1 mmol), stirred at 70 °C for 0.5 h, cooled to 23 °C, diluted with Et_2_O (100 mL) and H_2_O (50 mL). The two layers were separated and the aqueous layer was extracted with Et_2_O (6 × 50 mL). The combined organic layers were washed twice with a Na_2_S_2_O_3_ sat. aqueous solution (100 + 50 mL), dried over MgSO_4_, filtered, and evaporated to afford **4** (12.33 g, 93%) as a light yellow oil that crystallizes upon cooling in a freezer.

*R*_f_ = 0.70 (SiO_2_; hexane; KMnO_4_); [α]_D_^20^ = −32.0° (*c* 1.02, CHCl_3_); ^1^H NMR (400 MHz, CDCl_3_): *δ* = 5.17 (br s, 1H), 3.44 (dd, *J* = 9.7, 2.5 Hz, 1H), 3.30 (dd, *J* = 9.7, 5.4 Hz, 1H), 2.49 (br s, 1H), 2.03–1.84 (m, 2H), 1.79 (m, 1H), 1.64 (m, 1H), 1.62 (s, 3H), 1.59–1.48 (m, 2H), 1.41 (m, 1H), 1.33–1.10 (m, 3H), 0.99 (d, *J* = 6.4 Hz, 3H), 0.97–0.89 (m, 2H), 0.87 ppm (d, *J* = 6.5 Hz, 3H); ^13^C NMR (101 MHz, CDCl_3_): *δ* = 135.8, 121.1, 46.0, 42.1, 37.7, 35.6, 34.8, 27.7, 26.8, 26.1, 25.9, 24.0, 20.2, 19.9, 19.0 ppm; IR (ATR): $\tilde{\nu }$ = 2906, 2867, 1447, 1433, 1377, 1303, 1291, 1254, 1227, 1191, 1171, 1159, 1110, 1031, 990, 957, 942, 924 cm^−1^; HR-ESI-MS: molecule not ionized in ESI; GC/MS: *m/z* (%): 332 (4, [*M*]^+^), 163 (100, [*M* – C_3_H_6_I]^+^), 121 (19), 107 (25), 105 (7).

#### 4.2.4 (1*R*,4*R*,4a*S*,8a*S*)-1-((R)-1-Bromopropan-2-yl)-4,7-dimethyl-1,2,3,4,4a,5,6,8a-octahydronaphthalene (**5**)

A solution of **2** (100 mg, 0.45 mmol) in CH_2_Cl_2_ (2.5 mL) was treated with PPh_3_ (236 mg, 0.90 mmol), cooled to 0 °C, treated with CBr_4_ (375 mg, 1.13 mmol), stirred at 0 °C for 1 h, and evaporated. The crude was absorbed on SiO_2_ and column chromatography (SiO_2_ pre-treated with hexane/Et_3_N 99:01; hexane) gave **5** (128 mg, quant.) as a colorless oil.

*R*_f_ = 0.60 (SiO_2_; hexane; KMnO_4_); [α]_D_^20^ = −16.1° (*c* 0.94, CHCl_3_); ^1^H NMR (400 MHz, CDCl_3_): *δ* = 5.17 (br s, 1H), 3.61 (dd, *J* = 9.9, 2.6 Hz, 1H), 3.49 (dd, *J* = 9.9, 5.6 Hz, 1H), 2.49 (br s, 1H), 2.02–1.74 (m, 4H), 1.70–1.48 (m, 3H), 1.63 (s, 3H), 1.42 (m, 1H), 1.35–1.18 (m, 2H), 1.05 (d, *J* = 6.5 Hz, 3H), 1.03–0.90 (m, 2H), 0.87 ppm (d, *J* = 6.5 Hz, 3H); ^13^C NMR (101 MHz, CDCl_3_): *δ* = 135.7, 120.7, 44.2, 42.5, 42.1, 37.6, 35.8, 35.6, 27.7, 26.8, 26.1, 25.9, 24.0, 19.9, 17.0 ppm; IR (ATR): $\tilde{\nu }$ = 2907, 2869, 1447, 1435, 1379, 1334, 1306, 1290, 1261, 1236, 1218, 1185, 1159, 1140, 1110, 1075, 1037, 991, 958, 942, 925 cm^−1^; HR-ESI-MS: molecule not ionized in ESI; GC/MS: *m/z* (%): 286 (4, [*M*]^+^), 284 (4, [*M*]^+^), 163 (100, [*M* – C_3_H_6_Br]^+^), 121 (18), 107 (22), 105 (5).

#### 4.2.5 (1*R*,4*R*,4a*S*,8a*R*)-4,7-Dimethyl-1-(prop-1-en-2-yl)-1,2,3,4,4a,5,6,8a-octahydronaphthalene (amorpha-4,11-diene, **AD**)

##### 4.2.5.1 Microwave-assisted approach

Iodo derivative **4** (11.85 g, 35.7 mmol) was treated with *t-*BuOK (1 M in THF) (46.4 mL, 46.4 mmol), stirred at 65 °C in a microwave oven for 30 min, diluted with Et_2_O (150 mL) and H_2_O (150 mL). The two layers were separated and the aqueous layer was extracted with Et_2_O (4 × 75 mL). The combined organic layers were washed brine, dried over MgSO_4_, filtered, and evaporated. Column chromatography (SiO_2_; hexane) gave **AD** (7.03 g, 96%) as a colorless oil.

##### 4.2.5.2 Thermal approach

A solution of iodo derivative **4** (6.46 g, 19.43 mmol) in melted *t*-BuOH (13 mL) was treated with *t*-BuOK (4.4 g, 39.2 mmol), stirred at reflux for 3.5 h, diluted with Et_2_O (60 mL) and H_2_O (60 mL). The two layers were separated and the aqueous layer was further extracted with Et_2_O (4 × 30 mL). The combined organic layers were dried over MgSO_4_, filtered, and evaporated. Column chromatography (SiO_2_; cyclohexane) gave **AD** (2.52 g, 63%) as a colorless oil.

*R*_f_ = 0.77 (SiO_2_; hexane; KMnO_4_); *R*_f_ = 0.34 (RP-18; MeOH; KMnO_4_); [α]_D_^20^ = −13.9° (*c* 1.00, CHCl_3_) ([18]: [α]_D_^20^ = −14.0° (*c* 0.4, CHCl_3_)); ^1^H NMR (400 MHz, CDCl_3_): *δ* = 5.06 (br s, 1H), 4.87 (br s, 1H), 4.64 (br s, 1H), 2.55 (br s, 1H), 2.06–1.83 (m, 3H), 1.77 (m, 1H), 1.74 (s, 3H), 1.67 (dq, *J* = 12.9, 3.5 Hz, 1H), 1.60 (s, 3H), 1.59–1.46 (m, 2H), 1.40 (m, 1H), 1.35–1.18 (m, 2H), 0.98 (qd, *J* = 12.8, 3.4 Hz, 1H), 0.89 ppm (d, *J* = 6.4 Hz, 3H); ^13^C NMR (101 MHz, CDCl_3_): *δ* = 148.5, 135.1, 121.3, 110.2, 48.1, 42.3, 38.1, 35.9, 28.3, 26.9, 26.5, 26.3, 24.1, 23.1, 20.3 ppm; IR (ATR): $\tilde{\nu }$ = 2918, 2866, 1643, 1446, 1376, 1240, 1173, 1139, 1108, 993, 968, 936 cm^−1^; HR-ESI-MS: molecule not ionized in ESI; GC/MS: *m/z* (%): 204 (45, [*M*]^+^), 189 (73, [*M* – CH_3_]^+^), 119 (100). Analytical data correspond to the literature [18].
