# Supplementary material for: Synthesis of amorpha-4,11-diene from dihydroartemisinic acid
Source: Tetrahedron. 2019 Feb 8;75(6):743–8. doi: 10.1016/j.tet.2018.12.050 (PMC6346372; doi:10.1016/j.tet.2018.12.050)
Supplement: Multimedia component 1 [file mmc1.docx]

**Supporting Information**

Synthesis of Amorpha-4,11-diene from Dihydroartemisinic Acid

Geoffrey Schwertz^a,b^, Andrea Zanetti^a,b^, Marllon Nascimento^a,b^, Mario Andrès Gomez Fernandez^a,b^, Fabienne Dioury^b^, Janine Cossy^a,^*, Zacharias Amara^b,^*

*^a^ Laboratoire de Chimie Organique, Institute of Chemistry, Biology and Innovation (CBI), UMR 8231, ESPCI ParisTech/CNRS/PSL Research University, Paris Cedex 05, France*

*^b^ Equipe de Chimie Moléculaire, Laboratoire GBCM, EA7528, Conservatoire national des arts et métiers, HESAM Université, 2 rue Conté, Paris Cedex 03, France*

**Table of Contents**

[S1. GC/MS Spectra S3](#_Toc529390724)

[S2. NMR Spectra S8](#_Toc529390725)

# GC/MS Spectra

Figure S1. GC/MS spectra of 2.

Figure S2. GC/MS spectra of 3.

Figure S3. GC/MS spectra of 4.

Figure S4. GC/MS spectra of 5.

Figure S5. GC/MS spectra of AD.

# NMR Spectra

This Section contains NMR spectra of all compounds reported in this manuscript.

Figure S6. ^1^H NMR (400 MHz) spectra of compound 2 in CDCl_3_ at 298 K.

Figure S7. ^13^C NMR (101 MHz) spectra of compound 2 in CDCl_3_ at 298 K.

Figure S8. ^1^H NMR (400 MHz) spectra of compound 3 in CDCl_3_ at 298 K.

Figure S9. ^13^C NMR (101 MHz) spectra of compound 3 in CDCl_3_ at 298 K.

Figure S10. ^1^H NMR (400 MHz) spectra of compound 4 in CDCl_3_ at 298 K.

Figure S11. ^13^C NMR (101 MHz) spectra of compound 4 in CDCl_3_ at 298 K.

Figure S12. ^1^H NMR (400 MHz) spectra of compound 5 in CDCl_3_ at 298 K. Peak at 6.83 ppm corresponds to residual CHBr_3_.

Figure S13. ^13^C NMR (101 MHz) spectra of compound 5 in CDCl_3_ at 298 K.

Figure S14. ^1^H NMR (400 MHz) spectra of AD in CDCl_3_ at 298 K.

Figure S15. ^13^C NMR (101 MHz) spectra of AD in CDCl_3_ at 298 K.
